# Supplementary material for: Fast room temperature lability of aluminosilicate zeolites
Source: Nat Commun. 2019 Oct 16;10:4690. doi: 10.1038/s41467-019-12752-y (PMC6795794; doi:10.1038/s41467-019-12752-y)
Supplement: Supplementary file 1 — Supplementary Information [file 41467_2019_12752_MOESM1_ESM.pdf]

Fast Room Temperature Lability of Aluminosilicate Zeolites

Heard et al

Supplementary Information

Supplementary Figure 1.	^{17}O MAS NMR spectra of slurries of Al-CHA and $H_2{}^{17}O$
Supplementary Figure 2.	High-field 17O MQMAS NMR spectrum of a slurry of Al-CHA and $H_2{}^{17}O$
Supplementary Table 1.	NMR parameters extracted from high-field ^{17}O MQMAS NMR spectrum
Supplementary Figure 3.	^{1}H MAS NMR spectra of slurries of Al-CHA and $H_2{}^{17}O$
Supplementary Figure 4.	Helmholtz free energy curves for 300K biased MD simulations
Supplementary Table 2.	Helmholtz free energy of transition states and products at 300 K/450 K
Supplementary Figure 5.	Schematic for collective variables in free energy calculations.
Supplementary Figure 6.	Schematic for Brønsted proton solvation in H-CHA/H_2O

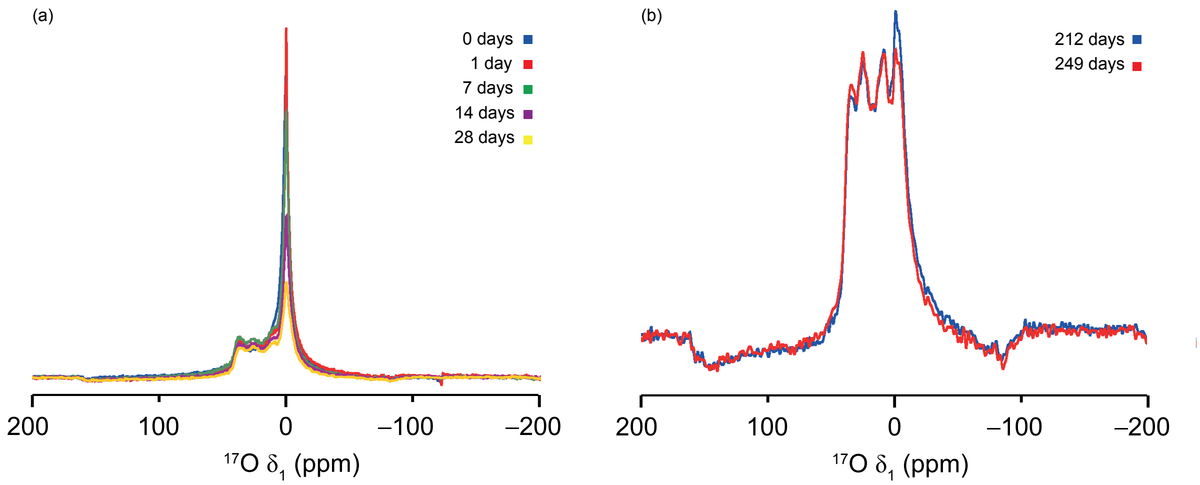

Supplementary Figure 1. ^{17}O (14.1 T, 10 kHz) MAS NMR spectra of calcined Al-CHA slurried in 40% $H_2{}^{17}O$, for varying times. Spectra were acquired using a pulse duration of (a) 0.5 μs and (b) 1.0 μs. Note there is no correlation between the absolute scale on the y axis between (a) and (b).

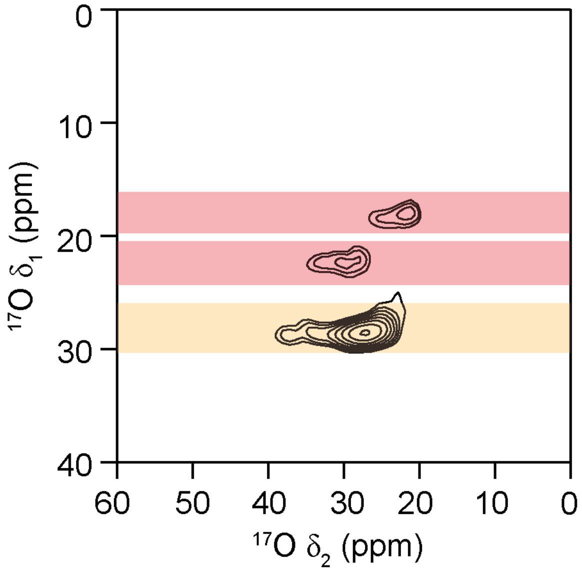

Supplementary Figure 2. ^{17}O (20.0 T, 10 kHz) 3QMAS NMR spectrum (shown after shearing) of calcined Al-CHA slurried in 40% $H_2{}^{17}O$ for 80 days. Regions highlighted in red are attributed to Si-^{17}O-Al species and those in gold to Si-^{17}O-Si species. Note spinning sidebands in the indirect dimension are not shown. NMR parameters extracted from this spectrum are given in Supplementary Table 1.

δ1	δ2	δiso	PQ	Identity

(ppm)	(ppm)	(ppm)	/ MHz	
18.1	21.8	28.9	4.0	Si-^{17}O-Al
22.4	29.7	36.7	3.9	Si-^{17}O-Al
28.5	31.5	44.4	5.3	Si-^{17}O-Si

Supplementary Table 1. NMR parameters extracted from the ^{17}O 3QMAS spectrum shown in Supplementary Figure 2.

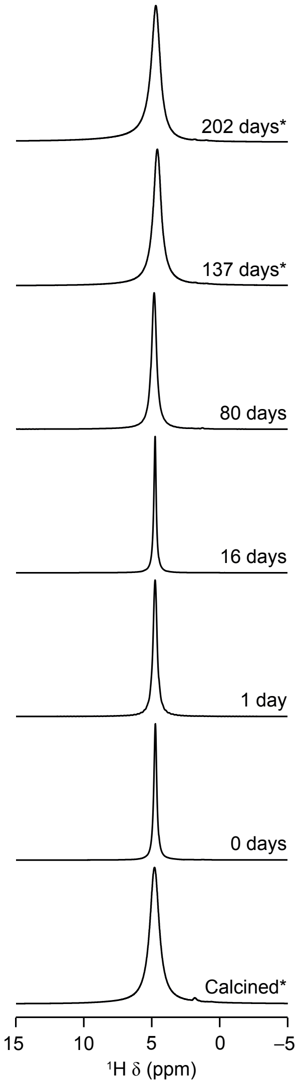

Supplementary Figure 3. ^{1}H (20.0 T, 10 kHz) MAS NMR spectra of slurries of calcined Al-CHA starting material and 40% $H_2{}^{17}O$. * denote spectra acquired at 9.4 T.

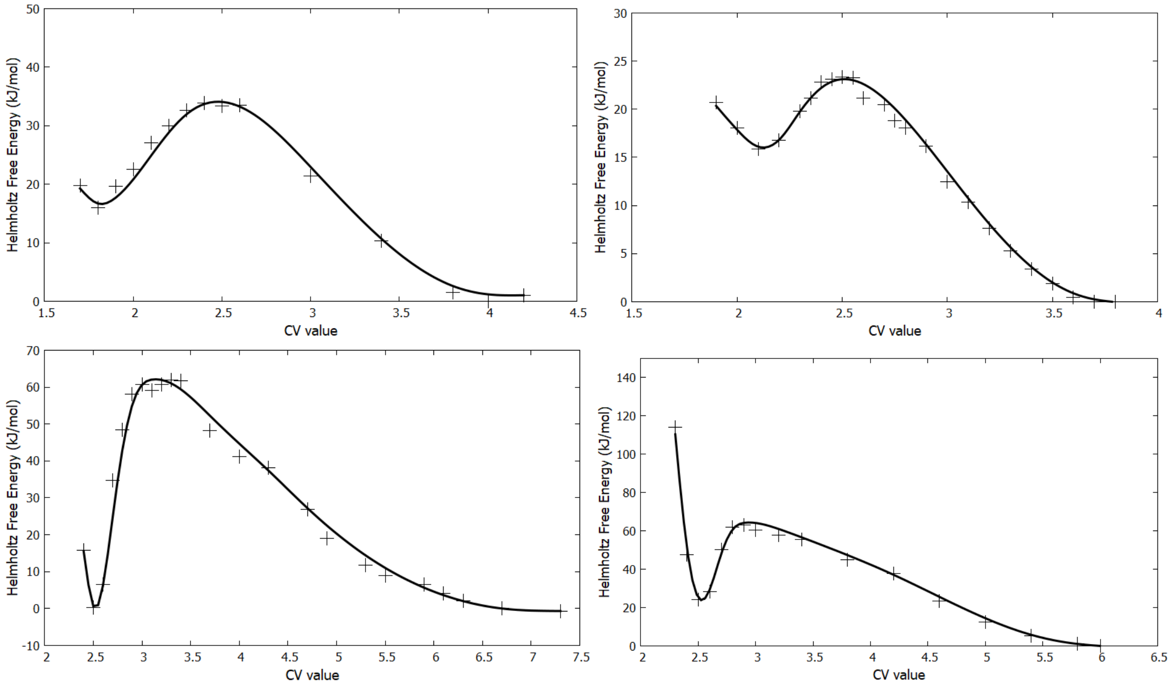

Supplementary Figure 4. Free energy profiles for reactions calculated at 300 K. Top left) H2O attack at O1 site of Al-O(H)-Si. Top right) H2O attack at O4 site of Al-O(H)-Si. Bottom left) Axial reaction at O1 site of Si-O-Si. Bottom right) Axial reaction at O4 site of Si-O-Si. Curves are a guide for the eye.

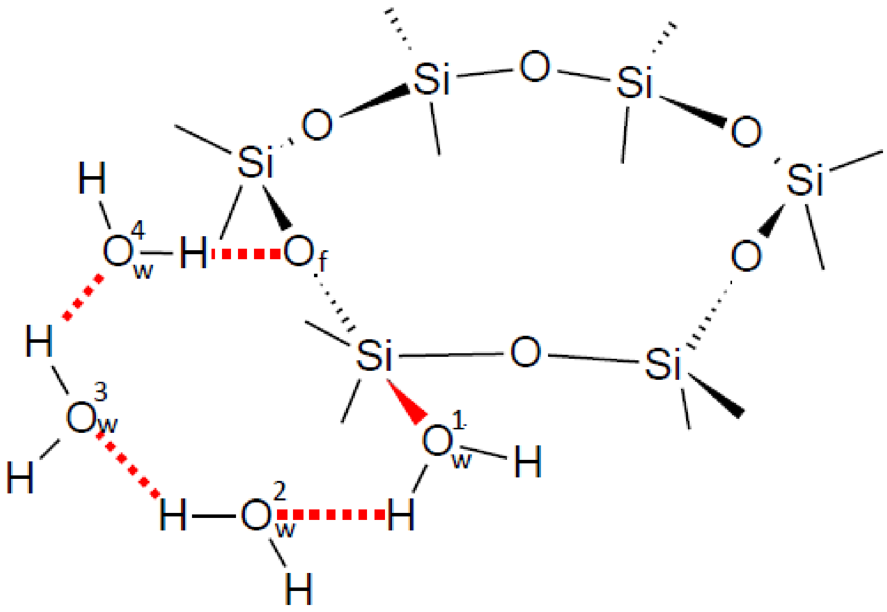

$$CV = \sqrt{\Sigma(r_{Si\,-\,O_w^1})^2 + (r_{H_w^1\,-\,O_w^2})^2 + (r_{H_w^2\,-\,O_w^3})^2 + (r_{H_w^3\,-\,O_w^4})^2 + \left(r_{H_w^4\,-\,O_f}\right)^2}$$

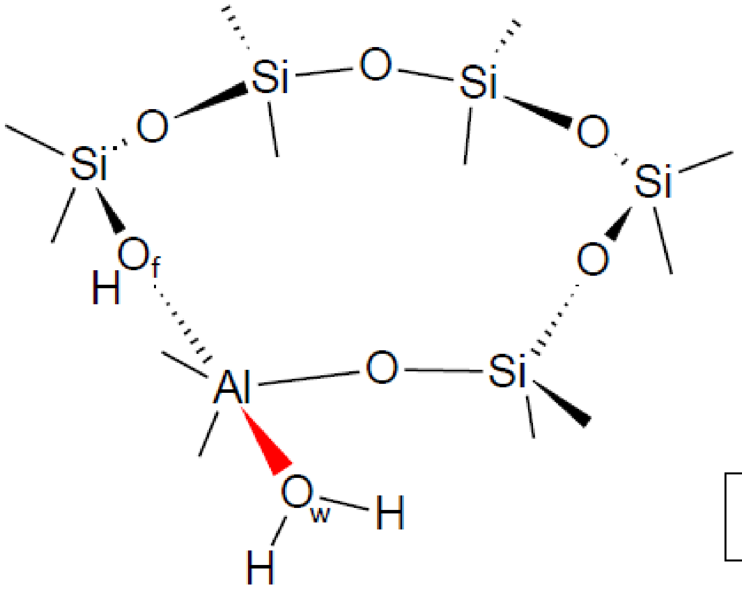

$$CV = r_{O_w-Al}$$

Supplementary Figure 5. Schematic depiction of the collective variables chosen for the Si-O and Al-O bond scission steps in CHA. Bonds involved in the CV are shown in red colour.

	Al-O-Si				Si-O-Si			
	O1		O4		O1		O4	
	$\Delta A^{\ddagger}$	ΔA_{rxn}	$\Delta A^{\ddagger}$	ΔA_{rxn}	$\Delta A^{\ddagger}$	ΔA_{rxn}	$\Delta A^{\ddagger}$	ΔA_{rxn}
300 K	32	17	24	16	63	1	63	24
450 K	38	22	25	15	85	10	80	34

Supplementary Table 2. Thermodynamic data calculated for 300K and 450K with ab initio biased molecular dynamics. For 450 K data with Al-O (O1), Al-O (O4), Si-O (O1), and Si-O (O4), free energy profiles are calculated with 32, 27, 27 and 26 points, respectively. The collective variables are unchanged between 300 K and 450 K simulations.

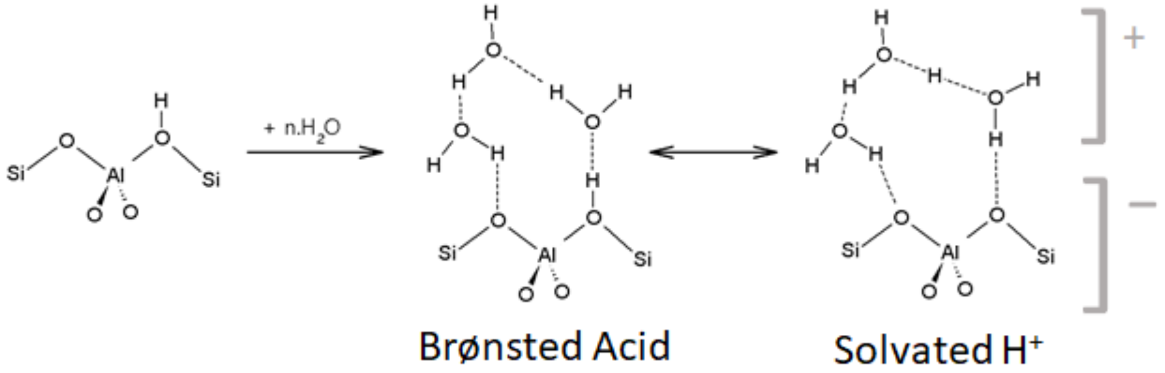

Supplementary Figure 6. A schematic of the equilibrium set up between a Brønsted proton localized on the zeolite framework and a Brønsted proton solvated by water in the zeolite pore. *Ab initio* equilibrium MD simulations at 300 K within the NVT ensemble using a Nosé-Hoover thermostat show a strong propensity towards solvated H^+. The following definition of solvation was applied to analyze the equilibrium: A configuration is considered to include an unsolvated proton, if the proton is closer to any framework oxygen (O_f) than to any of the water oxygen atoms (O_w) in the cell. If this is not true, the proton is said to be solvated. We find solvated states for > 99 % of frames in the MD trajectory.

Movies for approximate free energy pathways between reactants and products for the first hydrolysis step under full water loading conditions at AlO1 and SiO1, as approximated by slow-growth pathways. These files are available electronically.

For AlO1, a water molecule interacts with an Al atom through the water oxygen (Ow), which breaks the Al-O1 bond, leading to inversion of the Al tetrahedral centre, and abstraction of the Brønsted acidic proton from the solvation environment in the zeolite pore.

For SiO1, a water molecule interacts with a silicon atom through the water oxygen (Ow). A chain of water molecules shuttles a proton via a Grotthuss mechanism, to attach to a framework oxygen atom in an axial position. This breaks the framework Si-O1 bond, inverting the Si tetrahedral centre, creating two silanol groups.
