# Supplementary material for: Fast room temperature lability of aluminosilicate zeolites
Source: Nat Commun. 2019 Oct 16;10:4690. doi: 10.1038/s41467-019-12752-y (PMC6795794; doi:10.1038/s41467-019-12752-y)
Supplement: Supplementary file 3 — Description of Additional Supplementary Files [file 41467_2019_12752_MOESM3_ESM.docx]

**Description of Additional Supplementary Files**

**File Name: Supplementary Movie 1**

**Description:** Movie for approximate free energy pathways between reactants and products for the first hydrolysis step under full water loading conditions at SiO1, as approximated by slowgrowth pathways. For SiO1, a water molecule interacts with a silicon atom through the water oxygen (Ow). A chain of water molecules shuttles a proton via a Grotthuss mechanism, to attach to a framework oxygen atom in an axial position. This breaks the framework Si-O1 bond, inverting the Si tetrahedral centre, creating two silanol groups.

**File Name: Supplementary Movie 2**

**Description:** Movie for approximate free energy pathways between reactants and products for the first hydrolysis step under full water loading conditions at AlO1, as approximated by slowgrowth pathways. For AlO1, a water molecule interacts with an Al atom through the water oxygen (Ow), which breaks the Al-O1 bond, leading to inversion of the Al tetrahedral centre, and abstraction of the Brønsted acidic proton from the solvation environment in the zeolite pore.
